# Perimenstrual repetitive greater occipital nerve blockade for menstrual migraine: a randomized, double-blind, placebo-controlled trial

**DOI:** 10.1186/s12883-026-05238-0

**Published:** 2026-08-18

**Authors:** Okan Akşahin, Nazlı Gamze Bülbül, Mehmet Fatih Özdağ

**Affiliations:** 1 Department of Neurology, Gaziosmanpaşa Research and Training Hospital, Karayolları, Osmanbey 621st Street, Gaziosmanpaşa, Istanbul, 34255 Türkiye; 2 Department of Neurology, Sultan Abdulhamid Han Research and Training Hospital, Istanbul, Türkiye

**Keywords:** Menstrual migraine, Greater occipital nerve block, Peripheral nerve block, Migraine prophylaxis, Neuromodulation

## Abstract

**Background:**

The main purpose of this study is to demonstrate the decrease in headache severity, pain duration and the number of painful days in a month after repetitive GON blockade applied with local anesthetic in menstrual migraine patients compared to the placebo group.

**Methods:**

This randomized, double-blind, placebo-controlled study included 48 menstrual migraine patients. 24 patients received medication (0.5% bupivacaine) and 24 received placebo (saline) GON blockade twice a month for three months. Baseline data (month 0) relied on retrospective recall, whereas post-treatment data (months 3 and 6) were collected prospectively using a paper-based headache diary. The primary outcome was the change in headache severity (VAS score). Secondary outcomes included attack frequency, duration, number of headache days, analgesic use, and MIDAS scores.

**Results:**

Baseline characteristics were similar between groups. At month 3, the active group showed a significantly greater reduction in the primary outcome, VAS scores, compared to the placebo group (decreasing from 8.75 ± 0.67 to 3.80 ± 3.18 vs. 8.71 ± 1.04 to 7.16 ± 1.31; *p* < 0.001). Significant reductions were also observed in secondary outcomes including monthly attack frequency (*p* = 0.002), mean attack duration (*p* < 0.001), number of headache days (*p* = 0.002), MIDAS scores (*p* = 0.003), and analgesic use (*p* = 0.009). Mild, transient injection-site tenderness was the only reported side effect. The clinical improvements were maintained at the 6th month, suggesting a potential sustained benefit, though natural history or late placebo responses cannot be fully excluded.

**Conclusion:**

Repetitive perimenstrual GON blockade may be an effective short-term prophylaxis for MM. However, the observed effects on the subjective primary endpoint (VAS) may be partially attributable to imperfect blinding, and all secondary outcomes are exploratory. Further studies are needed to confirm these findings and exclude natural history or placebo responses.

**Trial registration:**

ClinicalTrials.gov, NCT07576309. Registered on 08 May 2026. Retrospectively registered.

**Supplementary Information:**

The online version contains supplementary material available at https://doi.org/10.1186/s12883-026-05238-0.

## Background

Migraine is a common primary headache disorder in the general population and is more frequently observed in women than in men [1]. Although the exact cause of this sex-dependent prevalence remains unknown, it is thought to be associated with fluctuations in estrogen and progesterone levels [2]. Menstrual migraine (MM) is one of the most frequently encountered migraine subtypes in the neurology outpatient clinic, affecting 60% of women with migraine, and its management frequently challenges both the physician and the patient [3, 4]. The International Headache Society has defined two types of MM: pure menstrual migraine (PMM) and menstrually related migraine (MRM) [3]. PMM presents with attacks occurring exclusively during the period of menstrual bleeding, whereas MRM is characterized by attacks that can occur at any time during the menstrual cycle, depending on the hormonal changes triggered by menstrual bleeding [3]. The treatment of MM is divided into three categories: acute treatment, short-term prophylaxis, and long-term prophylaxis. There are no licensed drugs specifically for the prophylaxis of menstrual migraine; however, short-term prophylaxis can be recommended for women who have a predictable relationship between migraine and menstruation [4]. Interventional treatments are also utilized in cases where there is no response to prophylactic medical treatments [5]. In patients with an inadequate response despite numerous medical treatments, blockade therapies offer an alternative solution due to their ease of administration, safety, and high cost-effectiveness compared to treatments such as botulinum toxin injections.

Considering the common pathophysiological mechanisms between migraine and MM, greater occipital nerve (GON) blockade may contribute to the indirect regulation of estrogen’s effects on calcitonin gene-related peptide (CGRP) by providing CGRP modulation, and it may be effective in MM prophylaxis. Furthermore, GON blockade can decrease peripheral nerve excitability by reducing hormonal sensitivity and can significantly limit the impact of hormonal fluctuations on the trigeminovascular system [5]. However, to date, there is an insufficient number of studies evaluating the efficacy of GON blockade in the treatment of MM [6]. Therefore, the present study aims to fill this important gap in the literature. The primary objective of the study is to demonstrate whether repetitive GON blockade administered with a local anesthetic in patients with MM significantly reduces headache severity, attack duration, and the number of pain days within a month compared to a placebo group.

The main hypothesis of this study is that repetitive GON blockade with a local anesthetic in patients with MM will provide a significant reduction in headache severity, attack duration, and the number of pain days within a month compared to the placebo group. To test this hypothesis, we designed a double-blind, randomized, placebo-controlled study to evaluate the efficacy of repetitive GON blockade in patients with MM. In the literature, a single prospective, open-label study examining the efficacy of GON blockade in MM reported significant improvements in pain severity, attack frequency, and quality of life following treatment [6]. Our study, on the other hand, is important as it is the first placebo-controlled trial in the literature aimed at evaluating the prophylactic efficacy of GON blockade in MM, which is a refractory migraine subtype.

## Methods

### Study design and participants

This prospective, randomized, double-blind, placebo-controlled clinical trial was conducted to investigate the efficacy and safety of repetitive bilateral greater occipital nerve (GON) blockade in women with menstrual migraine. The study protocol was approved by the institutional ethics committee on 26 September 2024, and written informed consent was obtained from all participants prior to their inclusion in the study. The trial was retrospectively registered on ClinicalTrials.gov (NCT07576309) on 08 May 2026 due to administrative delays in completing the online registration process, despite prior ethical approval and study initiation. We confirm that the public registry record is publicly accessible and that the registered primary and secondary outcomes match exactly those reported in this manuscript, with no discrepancies.

Female patients aged between 18 and 50 years who were diagnosed with pure menstrual migraine or menstrually related migraine according to the International Classification of Headache Disorders, 3rd edition (ICHD-3) diagnostic criteria were included in the study. As an inclusion criteria, a documented history of migraine attacks during the perimenstrual period (between days − 2 and + 3 relative to the onset of menstruation) in at least two of the last three menstrual cycles was required.

Patients with a diagnosis of chronic migraine, those with an additional primary or secondary headache disorder, those who were pregnant or lactating, those with a known hypersensitivity to local anesthetics, those who had received an occipital nerve blockade within the last three months, or those with a significant neurological or systemic disease that could affect the outcome evaluation were excluded from the study. Participants were excluded if they were currently using other prophylactic migraine medications. Throughout the study, patients maintained a paper-based prospective headache diary to record attack characteristics and the use of acute rescue analgesics.

A total of 48 patients were randomized in a 1:1 ratio and assigned to either the active treatment (0.5% bupivacaine) or placebo (saline) group, with 24 participants in each group. This study was conducted and reported in adherence to the Consolidated Standards of Reporting Trials (CONSORT) guidelines.

### Randomization and blinding

The preparation of active drug and placebo formulations was carried out under Good Manufacturing Practices (GMP) conditions at the facilities of the Faculty of Pharmacy Technology Development Zone. As the active treatment, 0.5% bupivacaine was used, while saline was used as the placebo. Blinding was ensured by preparing the formulations to be indistinguishable in terms of appearance, volume, and labeling. Randomization was performed using a computer-generated random number sequence. To ensure allocation concealment, treatment assignments were kept in sequentially numbered, opaque, sealed envelopes. The sequence was generated and concealed by an independent researcher not involved in patient care or evaluation. The investigational pharmacist prepared the identical syringes based on this sequence. The treatment allocation was known only to the investigational pharmacist and the study coordinator, keeping the patients, administering physicians, and outcome assessors completely blind throughout the study.

### Intervention protocol

Participants received bilateral GON blockade twice a month for three months, and each patient received a total of six injections. Injections were administered seven days before the expected onset of menstruation and on the first day of menstruation in each cycle.

The active treatment group received 0.5% bupivacaine, whereas the placebo group received an equivalent volume of saline. All injections were performed by an experienced neurologist using a standard anatomical landmark technique. Corticosteroids were not used during the procedure.

### Outcome measures

Evaluations were conducted at baseline (month 0), at the end of the intervention period (month 3), and at the follow-up visit (month 6).

The primary outcome measure was the change in headache severity evaluated using the Visual Analog Scale (VAS) [7, 8].

The baseline VAS scores evaluated during the interictal period reflect the patients’ retrospective assessment of the average severity of their characteristic menstrual migraine attacks experienced during their preceding menstrual cycles, rather than an active attack at the time of evaluation. It is important to note that the baseline VAS was based on the patients’ retrospective recall of their characteristic attacks, whereas the month-3 and month-6 values were recorded prospectively using the headache diary.

Secondary outcome measures included monthly migraine attack frequency, number of headache days per month, mean attack duration, monthly analgesic consumption, and Migraine Disability Assessment (MIDAS) [9] scores.

Participants maintained a prospective headache diary throughout the study to record attack characteristics and medication use.

### Statistical analysis

Normality of data distribution was evaluated using the Shapiro-Wilk test. Since the variables did not show a normal distribution, non-parametric tests were utilized. Between-group comparisons utilized raw follow-up values with the Mann-Whitney U test. Because baseline values were statistically balanced across both arms, this unadjusted comparison was a deliberate choice for this exploratory pilot study, rather than a statistical impossibility for baseline-adjusted analysis. Within-group changes across baseline, 3rd month, and 6th month were evaluated using the Friedman test. Treatment effects were interpreted based primarily on inter-group comparisons rather than solely on intra-group changes to reduce regression to the mean bias. All statistical analyses were performed using IBM SPSS Statistics, version 22.0. All secondary outcomes were defined a priori but are interpreted as exploratory and hypothesis-generating. Due to the exploratory nature of the study, no formal correction for multiple comparisons (e.g., Bonferroni correction) was applied for the secondary outcomes. Given the exploratory nature of this pilot study, a formal a priori power calculation was not performed. The sample size was primarily based on clinical feasibility regarding this specific migraine subgroup. A two-sided p-value < 0.05 was considered statistically significant.

## Results

### Participant characteristics

All 48 randomized patients completed the study protocol and were included in the final analyses. At baseline, no statistically significant differences were found between the active and placebo groups in terms of demographic and clinical characteristics, including migraine frequency, attack severity, attack duration, number of headache days, analgesic consumption, and VAS and MIDAS scores. This indicates that the randomization was successful. All 48 participants (100%) reported using acute rescue analgesics for their migraine attacks prior to the intervention. The baseline monthly analgesic consumption did not differ significantly between the active (6.21 ± 2.92) and placebo (6.75 ± 2.90) arms (*p* = 0.448). Among our 48 participants, 7 patients were diagnosed with pure menstrual migraine (PMM) and 41 patients with menstrually related migraine (MRM). These subtypes were distributed similarly between the active and placebo groups, and no statistically significant differences in treatment response were observed between the two subtypes.

### Primary outcome measure: Visual Analog Scale (VAS)

At the third-month evaluation, a statistically significant decrease in VAS [7, 8] scores was detected in the active GON blockade group compared to the placebo group (*p* < 0.001) (Table 1) (Fig. 1a). The reduction in headache severity was clinically significant and beyond the change observed in the placebo group. This therapeutic effect was observed to be maintained at the sixth-month evaluation, suggesting a potential sustained benefit, though natural history or late placebo responses cannot be fully excluded.

**Table 1:** Within-group and between-group comparison of Visual Analog Scale (VAS) scores at baseline, 3 months, and 6 months (Primary Outcome)

Timepoint	Drug (Mean ± SD)	Drug (Median)	Placebo (Mean ± SD)	Placebo (Median)	U	*p*-value
Baseline	8.75 ± 0.67	9	8.71 ± 1.04	9	283.5	0.922
3 months	3.80 ± 3.18	5	7.16 ± 1.31	7	96.5	**< 0.001**
6 months	3.83 ± 3.18	5	7.50 ± 1.25	8	81.5	**< 0.001**
Within-group (*χ²*, *p*)†	*χ²* = 40.415	***p*** **< 0.001**	*χ²* = 32.411	***p*** **< 0.001**	-	-

*SD* Standard Deviation, *VAS* Visual Analog Scale, *U* Mann-Whitney U test, *χ²* Chi-square value

Significant *p*-values are highlighted in bold

†Friedman test was used for within-group comparisons

**Fig. 1:**
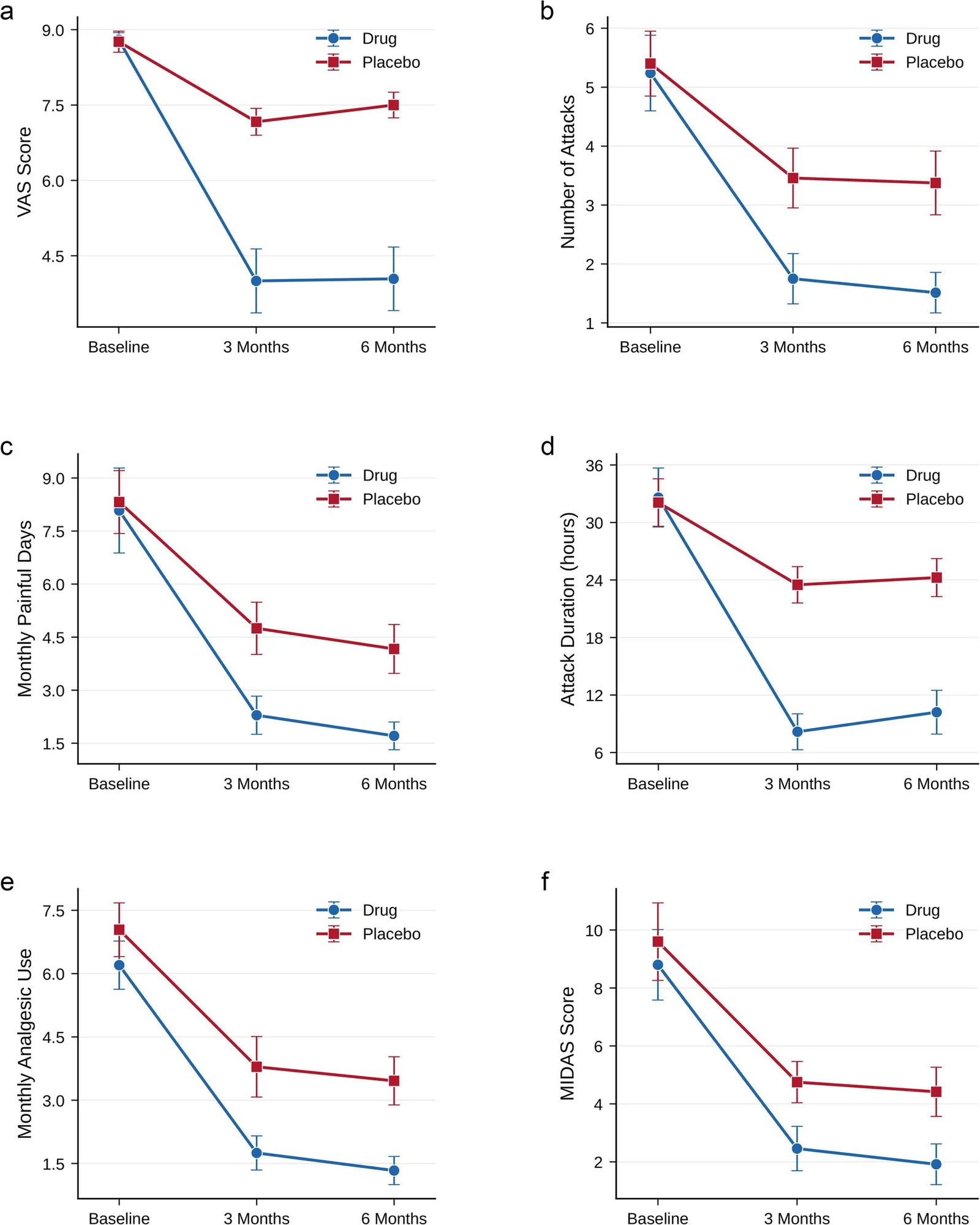
**a**: Change in headache severity after GON blockade. **b**: Change in the monthly number of attacks after GON blockade. **c**: Change in the monthly number of painful days after GON blockade. **d**: Change in attack duration after GON blockade. **e**: Change in the monthly number of analgesics used after GON blockade. **f**: Change in MIDAS scores after GON blockade

### Secondary outcome measures

For monthly migraine attack frequency at the third month, a significant difference was found between the groups in favor of the active treatment (*p* = 0.002) (Table 2) (Fig. 1b). The reduction in attack frequency was also maintained at the sixth-month follow-up evaluation, suggesting a continuity of the treatment effect.

**Table 2:** Within-group and between-group comparison of migraine attack numbers (monthly average) at baseline, 3 months, and 6 months (Secondary Outcome)

Timepoint	Drug (Mean ± SD)	Drug (Median)	Placebo (Mean ± SD)	Placebo (Median)	U	*p*-value
Baseline	5.12 ± 3.22	5	5.41 ± 2.81	5	269.0	0.691
3 months	1.71 ± 2.11	1	3.45 ± 2.48	3	141.5	**0.002**
6 months	1.50 ± 1.70	1	3.37 ± 2.65	3	140.5	**0.002**
Within-group (*χ²*, *p*)†	*χ²* = 29.293	***p*** **< 0.001**	*χ²* = 16.909	***p*** **< 0.001**	-	-

*SD* Standard Deviation, *U* Mann-Whitney U test, *χ²* Chi-square value

Significant *p*-values are highlighted in bold

†Friedman test was used for within-group comparisons

### Number of headache days per month

The decrease in the number of headache days per month in the active treatment group was significantly greater than in the placebo group (*p* = 0.002) (Table 3) (Fig. 1c). When evaluated over time, the clinical course diverged significantly between the two groups.

**Table 3:** Within-group and between-group comparison of the number of painful days (monthly average) measured at baseline, 3 months, and 6 months (Secondary Outcome)

Timepoint	Drug (Mean ± SD)	Drug (Median)	Placebo (Mean ± SD)	Placebo (Median)	U	*p*-value
Baseline	8.10 ± 6.13	6.5	8.25 ± 4.53	7.5	260.5	0.569
3 months	2.25 ± 2.67	1	4.75 ± 3.62	4	143.0	**0.002**
6 months	1.66 ± 1.95	1	4.16 ± 3.38	4	128.0	**0.001**
Within-group (*χ²*, *p*)†	*χ²* = 33.175	***p*** **< 0.001**	*χ²* = 20.575	***p*** **< 0.001**	-	-

*SD* Standard Deviation, *U* Mann-Whitney U test, *χ²* Chi-square value

Significant *p*-values are highlighted in bold

†Friedman test was used for within-group comparisons

### Mean attack duration

The mean attack duration significantly decreased in the active group at the third month compared to the placebo group (*p* < 0.001) (Table 4) (Fig. 1d). This effect was observed to continue at the sixth month, which may suggest a prolonged effect, though this should be interpreted with caution regarding the sustained influence of GON blockade on attack dynamics.

**Table 4:** Within-group and between-group comparison of mean attack durations (hours) at baseline, 3 months, and 6 months (Secondary Outcome)

Timepoint	Drug (Mean ± SD)	Drug (Median)	Placebo (Mean ± SD)	Placebo (Median)	U	*p*-value
Baseline	32.95 ± 15.62	24	31.41 ± 12.20	30	277.5	0.820
3 months	8.04 ± 9.28	6	23.50 ± 9.30	24	63.0	**< 0.001**
6 months	9.95 ± 11.35	8	24.25 ± 9.70	24	82.5	**< 0.001**
Within-group (*χ²*, *p*)†	*χ²* = 36.940	***p*** **< 0.001**	*χ²* = 13.153	***p*** **< 0.001**	-	-

*SD* Standard Deviation, *U* Mann-Whitney U test, *χ²* Chi-square value

Significant *p*-values are highlighted in bold

†Friedman test was used for within-group comparisons

### Monthly analgesic consumption

Monthly analgesic consumption significantly decreased in the active treatment group compared to the placebo group (*p* = 0.009) (Table 5) (Fig. 1e). This finding indicates not only symptomatic improvement but also a reduced need for rescue medications.

**Table 5:** Comparison of the number of analgesics used (monthly average) at baseline, 3 months, and 6 months, both within and between groups (Secondary Outcome)

Timepoint	Drug (Mean ± SD)	Drug (Median)	Placebo (Mean ± SD)	Placebo (Median)	U	*p*-value
Baseline	6.21 ± 2.92	6	6.75 ± 2.90	6	252.0	0.448
3 months	1.75 ± 1.98	1	3.80 ± 3.51	3	162.0	**0.009**
6 months	1.30 ± 1.65	1	3.45 ± 2.80	3	122.5	**0.001**
Within-group (*χ²*, *p*)†	*χ²* = 38.732	***p*** **< 0.001**	*χ²* = 20.321	***p*** **< 0.001**	-	-

*SD* Standard Deviation, *U* Mann-Whitney U test, *χ²* Chi-square value

Significant *p*-values are highlighted in bold.

†Friedman test was used for within-group comparisons

### MIDAS scores

Migraine-related disability, when evaluated with MIDAS [9], significantly decreased in the active group compared to the placebo group (*p* = 0.003) (Table 6) (Fig. 1f). This reduction demonstrates that an improvement was achieved in functional outcomes beyond pain parameters.

**Table 6:** Within-group and between-group comparison of Migraine Disability Assessment (MIDAS) scores at baseline, 3 months, and 6 months (Secondary Outcome)

Timepoint	Drug (Mean ± SD)	Drug (Median)	Placebo (Mean ± SD)	Placebo (Median)	U	*p*-value
Baseline	8.80 ± 6.21	6	8.75 ± 5.27	8.5	247.5	0.392
3 months	2.41 ± 3.77	1	4.75 ± 3.50	4	144.0	**0.003**
6 months	1.91 ± 3.43	0	4.41 ± 4.16	3.5	153.0	**0.004**
Within-group (*χ²*, *p*)†	*χ²* = 41.663	***p*** **< 0.001**	*χ²* = 22.615	***p*** **< 0.001**	-	-

*SD* Standard Deviation, *MIDAS* Migraine Disability Assessment, *U* Mann-Whitney U test, *χ²* Chi-square value

Significant *p*-values are highlighted in bold

†Friedman test was used for within-group comparisons

### Safety

No serious adverse events were reported during the study period. Side effects were mild and transient, primarily limited to local tenderness at the injection site. No participant discontinued the study due to a treatment-related adverse event.

## Discussion

In this randomized, double-blind, placebo-controlled study, it was demonstrated that repetitive bilateral greater occipital nerve (GON) blockade scheduled for the perimenstrual period provided significant and sustained improvement in headache severity, attack frequency, number of headache days, attack duration, analgesic consumption, and migraine-related disability in women with menstrual migraine. Furthermore, it is noteworthy that this clinical improvement continued during the three-month medication-free follow-up period after the completion of the active injection phase. This study is the first randomized, double-blind, placebo-controlled trial evaluating the efficacy of GON blockade deliberately scheduled for the perimenstrual period in the menstrual migraine subgroup. In this respect, our study fills a significant gap regarding the targeted peripheral neuromodulation approach for menstrual migraine.

The prevalence of migraine is approximately 2 to 3 times higher in women than in men, and attack frequency may increase, particularly in women of reproductive age, due to the effects of hormonal changes [10, 11]. Menstrual migraine (MM) is a specific migraine subtype triggered during the menstrual period due to a sudden drop in estrogen levels, characterized by longer, more severe, and more treatment-resistant attacks [12]. It is known that estrogen withdrawal sensitizes the trigeminovascular system and facilitates neuropeptide release [10, 13]. The detection of elevated CGRP levels during the menstrual period supports this biological mechanism. Functional neuroimaging and clinical studies have shown that GON blockade modulates nociceptive transmission at the level of the trigeminocervical complex (TCC) [14]. Furthermore, a significant decrease in serum CGRP levels has been reported following GON blockade [14, 15]. Local anesthetics are known to reduce pain transmission through unmyelinated C-fibers by blocking voltage-gated sodium channels [16]. However, it is suggested that the long-term effect of GON blockade does not depend solely on the duration of the local anesthetic; it may induce sustained modulation in central pain pathways through peripheral afferent inhibition [17, 18]. In this context, it can be considered that GON blockade timed to the perimenstrual period may reduce the increased trigeminovascular sensitivity associated with estrogen withdrawal and raise the threshold in the trigeminovascular system [13]. However, because serum CGRP and estrogen levels were not biochemically measured in our study, this proposed pathophysiological mechanism must be considered strictly speculative.

Current short-term prophylaxis strategies in the treatment of menstrual migraine include triptans, non-steroidal anti-inflammatory drugs, and hormonal regulations [19]. However, these approaches entail issues such as systemic drug burden, potential for side effects, and inadequate response in some patients [19]. The model applied in this study, on the other hand, is a protocol involving two injections per month for three months, the effects of which continue during the subsequent medication-free period. This suggests that GON blockade can be evaluated as a non-systemic, short-term, and targeted prophylactic strategy.

Although the efficacy of GON blockade has been evaluated in various studies for chronic migraine, cluster headache, and other primary headaches, randomized controlled trial data specifically for MM are not yet available [20–22]. In the prospective, open-label study by Cetin et al., 33 patients diagnosed with MM (15 pure menstrual migraine [PMM], 18 menstrually related migraine) received bilateral GON blockade once a month for three months; significant improvements were detected in VAS (Visual Analog Scale), MIDAS (Migraine Disability Assessment), and BDI (Beck Depression Inventory) scores in both subgroups, and no significant difference was observed between the groups. It was reported that this improvement also continued during the medication-free follow-up period (months 3–6) [6]. Our current study yields parallel results with this study in terms of the efficacy of repetitive GON blockade treatment in patients with MM. The original aspect of our study is that it was applied in a specific subgroup associated with hormonal triggering rather than the general migraine population, and utilized a short-term prophylaxis model timed to the perimenstrual period instead of acute treatment. Additionally, a repetitive protocol synchronized with cyclic and hormonal fluctuations was preferred over a single blockade application. This approach also contributes to the application strategies of GON blockade, which have not yet been standardized in the literature [20]. While GON blockade is an established intervention recommended for various migraine subtypes independent of hormonal triggers, our study highlights that synchronizing the blockade specifically to the perimenstrual period creates a highly effective, targeted short-term prophylaxis for menstrual migraine.

In our study, improvements in clinical parameters were also observed in the placebo group. It is known that the placebo effect in migraine treatment can be more pronounced, particularly with invasive procedures [23]. Interestingly, however, the placebo-group response regarding the primary pain endpoint (VAS, decreasing from 8.71 to 7.16) was relatively modest for such an invasive procedure. This limited placebo effect on pain intensity might reflect the uniquely severe, prolonged, and refractory nature of perimenstrual migraine attacks. In a related study by Gul et al., improvement was observed in the placebo group during the early period, but it was reported that the effect did not persist in the subsequent months [24]. In contrast, the persistence of improvement in the placebo group in our study raises the possibility of modulation on the trigeminocervical complex due to the mechanical pressure effect of the injection [18]. Nevertheless, the superiority of the active group over the placebo in the double-blind design and multiple clinical outcome measures demonstrates that the observed effect cannot be explained solely by the placebo.

From a clinical perspective, there is not only statistical significance but also clinical significance. Reductions in attack frequency, duration, and severity; the decrease in analgesic use; and the prominent improvement in MIDAS scores indicate that patients achieved significant gains in functionality and quality of life. Considering the impact of migraine on workforce loss and social life [25, 26], these findings are important for clinical practice.

Regarding the safety profile, only a local anesthetic was used in our study, and no serious adverse events were observed. Side effects were limited to mild and transient local reactions. It is also reported in the literature that GON blockade is generally a safe procedure [20]. The non-use of steroids provides a significant advantage in terms of long-term side effect risks [27].

The strengths of the study include the randomized, double-blind, and placebo-controlled design; the standardization of perimenstrual timing; prospective follow-up; and the medication-free observation period. However, there are several limitations. First, the study is single-center and the sample size is relatively limited, lacking an a priori formal power calculation. Second, because serum estrogen, progesterone, and CGRP levels were not biochemically measured, the proposed pathophysiological mechanism suggesting that GON blockade modulates estrogen withdrawal-triggered sensitization remains speculative. Third, regarding patient blinding, a topical anesthetic was not applied before the injections. The characteristic numbness induced by bupivacaine, which is absent with saline, may have influenced the effectiveness of patient blinding, representing a methodological limitation. Because we did not formally record allocation guessing at the end of the study, we cannot exclude the possibility that patients plausibly identified their allocation due to the lack of occipital numbness in the saline group. This imperfect blinding directly threatens the primary conclusion, as it may bias the subjective, patient-reported primary endpoint (VAS) in the direction of the observed effect. Fourth, the previous prophylactic treatment history of the participants was not systematically recorded, which constitutes another limitation. Finally, efficacy data beyond six months are not available, and the lack of correction for multiple comparisons should be taken into account when interpreting the secondary outcomes. Furthermore, while the reduction in monthly migraine days or attack frequency is the gold-standard primary outcome for migraine preventive trials, this study designated the VAS score as the primary outcome to specifically capture the profound pain severity characteristic of menstrual migraine attacks. Although this represents a methodological limitation in the context of standard prophylactic trial designs, the highly significant improvements observed in our secondary outcomes (number of headache days and attack frequency) robustly support the preventive efficacy of the intervention. Additionally, mixing measurement modes —relying on retrospective recall for baseline VAS while using prospective diaries for follow-up— introduces recall bias and potential regression to the mean, which may artificially inflate the apparent within-group reductions. Nevertheless, the observation of a consistent and sustained treatment effect across multiple clinical outcome measures supports the internal validity of the findings.

In conclusion, this study demonstrates that a targeted peripheral neuromodulation approach for a hormonally triggered migraine subtype in women with menstrual migraine can be effective and safe. Repetitive GON blockade scheduled for the perimenstrual period can be considered as a non-systemic, short-term prophylaxis strategy in patients with a predictable attack window and has the potential to be included in future menstrual migraine treatment algorithms.

## Conclusion

This study demonstrated that repetitive GON blockade might serve as an option for short-term prophylaxis in MM. While improvements were observed, the primary conclusion regarding subjective pain reduction must be interpreted cautiously due to potential imperfect blinding, and all secondary outcomes should be considered exploratory. Additionally, the persistence of benefit at month 6 cannot definitively exclude natural history or late placebo responses. To validate these findings and better elucidate the long-term effects of GON blockade, longer-term studies with larger cohorts are needed.

## Supplementary Information

Supplementary Material 1.

## Data Availability

The data supporting the findings of this study is available from the corresponding author upon reasonable request.

## Abbreviations

BDI: Beck Depression Inventory
CGRP: Calcitonin gene-related peptide
GMP: Good Manufacturing Practices
GON: Greater occipital nerve
ICHD-3: International Classification of Headache Disorders, 3rd edition
IHS: International Headache Society
MIDAS: Migraine Disability Assessment
MM: Menstrual migraine
MRM: Menstrually related migraine
PMM: Pure menstrual migraine
TCC: Trigeminocervical complex
VAS: Visual Analog Scale
